# 

**Published:** 1893-02

**Authors:** 


					﻿Vol. XIII.
FEBRUARY, 1893.
NO. .27
THE
HOMEOPATHIC pHY^lClA]\l,
A Monthly Journal of Homoeopathic Materia Medica
and Clinical Medicine.
"He is a freeman whom truth makes free, and all are slaves beside.”
“ Seek the Truth: come whence it may, cost what it will.”
EDITED AND PUBLISHED BY
WALTER M. JAMES, M. D.
PHILADELPHIA :
No. 1125 SPRUCE STREET.
X> O IT z> pur:
ALFRED HEATH & CO., Sole Agents fob Great Britain,
114 Ebury Street, Eaton Square.
Yearly Subscription, $2.50. invariably in advance. Single numbers, 25 ets.
Entered al the Philadelphia Poet-Offioe as Becood-Claaa Mall Matter.
				

